# The antibiotic prescribing behaviors of physicians are changed via rapid antigen test practice in the context of rational drug use

**DOI:** 10.3906/sag-1908-164

**Published:** 2020-06-23

**Authors:** Deniz UZUN, Halil KARA, Muhammed Fatih DOĞAN, Seyfullah Oktay ARSLAN

**Affiliations:** 1 Pharmaceuticals and Medical Devices Services Presidency, Ankara Provincial Health Directorate, Ankara Turkey; 2 Department of Pharmacology, Ankara Yıldırım Beyazıt University, Ankara Turkey; 3 Department of Pharmacology, Pamukkale University, Denizli Turkey

**Keywords:** Group A beta hemolytic streptococcus, family medicine, rapid antigen test, rational antibiotics use

## Abstract

**Background/aim:**

Rapid antigen test (RAT) is a practical test to detect the presence of Group A beta hemolytic streptococcus antigens in throat swab samples. The aim of this study is to investigate the changes in the empiric antibiotic prescribing behavior of 10 family physicians in Kırıkkale Province after using RAT in 2017.

**Materials and methods:**

RAT test practice started in Family Medicine in February 2017. Family Medicine Information System (FMIS) includes clinical and prescription records of 10 family physicians, providing health service to approximately 35,000 residents in Kırıkkale. The numbers of antibiotics prescribed by the physicians according to the ICD-10 codes (including upper respiratory tract infections) in February, March, and April of 2015, 2016, 2017 were determined. The number and group of antibiotics prescribed by the family physicians with the determined diagnosis and time periods were specified in the FMIS and recorded.

**Results:**

Antibiotic prescription behaviors of family physicians do not show a significant difference between 2015 and 2016. There was a dramatic and significant decrease in the number of prescribed antibiotics in 2017 compared to 2015 and 2016 (P < 0.05).

**Conclusion:**

This study shows that there has been a significant decrease in antibiotic prescription in 10 Family Medicine departments in 2017 in comparison to February, March, and April 2015 and 2016. The use of RAT resulted in a decrease in antibiotic prescription rates in 2017.

## 1. Introduction

In recent years, the maximum utility of drugs in medicine and minimization of its problems that may occur is an important issue on the agenda. The World Health Organization described rational drug use (RDU) in 1985. RDU is a set of rules that require the drugs to be taken at the most cost-effective way to meet the needs of the patient and the society at the appropriate time in accordance with the clinical needs. In order to eliminate the problems that arise due to the use of drugs, RDU should become widespread. All health workers, drug-related units, and citizens, especially physicians, have a significant responsibility [1]. The selection of appropriate medicine according to the diagnosis of the patient, prescription, follow-up of the treatment, informing the patient, training the medical staff have a key role for physicians on account of RDU. The physicians working in primary health care institutions have a special position and role in the implementation of RDU. Physicians, especially for antibiotics, should prevent excessive and incorrect medicine usage, economic losses, and adverse effects, and more importantly, for the reduction of antimicrobial resistance, should establish a correct diagnosis with the approaches in the context of RDU [2]. 

One of the microorganisms that cause disease in the upper respiratory tract is group A beta hemolytic streptococci (GAS). This bacterium is the cause of acute tonsillopharyngitis. Because of the large number of microorganisms that form acute tonsillopharyngitis (viral and other bacterial agents), this group of disease is the most common antibiotic prescribed misdiagnosis group. Rapid diagnosis and treatment are important in bacterial tonsillopharyngitis because of the possibility to cause complications in the course of the disease, especially in primary care [3,4]. Rapid antigen test (RAT) is used to diagnose GAS infections, which are among the agents of acute tonsillopharyngitis and cause serious side effects and complications [5]. In addition, antigen tests have been developed (such as influenza A and streptococcus pneumoniae) in order to enable rapid and accurate diagnosis against some microorganisms [6,7]. RAT is a practical test for detecting the presence of GAS antigen. Its sensitivity is partly low, but its specificity is very high. For clinical symptoms and infection, the positivity of RAT used to diagnose GAS is suitable for antibiotic prescription [8]. 

Antibiotics are among the most widely used drugs in Turkey, as in many countries. GAS should be treated with the appropriate antibiotic and dosage in the context of RDU since the irrational use of antibiotics result in the development of rapid resistance and leads to increase in complications and prolonged disease process [2,9,10].

The aim of this study is to compare the pharmacological data of 2017 with 2015 and 2016. In fact, Ministry of Health enacted the obligation of RAT procedure in 2017.

## 2. Materials and methods

### 2.1. Data collecting

This is a retrospective study of pediatric and adult patients admitted to outpatient clinics of ten family medicine units in which approximately 35,000 patients are registered in Kırıkkale Province. Our study was approved by Ankara Yıldırım Beyazıt University Yenimahalle Training and Research Hospital Clinical Research Ethics Committee (No: 2017/04). RAT application to avoid unnecessary use of antibiotics and to provide pharmacoeconomic efficiency started in family medicine units in February 2017 in Turkey. Therefore, Provincial Public Health Directorates distributed RATs to family physicians after giving RAT practice and evaluation training, in January 2017. When the use of RAT is required, the RAT outcome is entered into the Family Medicine Information System. If the result is positive, an antibiotic prescription is appropriate. If the result is negative, prescribing antibiotics is not suggested. The lists of patients who were diagnosed with International Classification of Diseases-10 (ICD-10 code) were accessed by entering the Family Medicine Information System (FMIS) from physicians’ computers in ten family medicine departments. The antibiotic names on the prescription pages of patients were found and noted in February, March, and April in 2015, 2016, and 2017. For 2017, both RAT-positive (+) and RAT-negative (–) results were recorded together with prescribed antibiotics. The age and sex of patients were also recorded.

### 2.2. RAT practice

RAT is the detection of carbohydrate antigen in the cell wall by using the enzyme immunoassay method. RAT has started to be used to make empirical antibiotic prescriptions suitable for rational medicine usage and pharmacoeconomically efficient. Primarily, the Ministry of Health trained the family physicians about the application and interpretation of the test. These tests were TOYO-brand in vitro diagnostic tests for the detection of group A beta-hemolytic streptococci (GAS). The regulation of FMIS, which is used by every physician, including the appropriate diagnostic criteria, was arranged simultaneously with the distribution of RAT kits to family physicians. Since the probability of GAS in the 0–3 age group was 0–6%, RAT was not required under 3 years of age. After the examination of the patients who were thought to be related to one of the diagnoses in the ICD-10 codes [11] (Table 1) determined by the Ministry of Health, they were allowed to see the table which was compatible with the modified Centor criteria [12] (Table 2) when the family physicians entered the FMIS together with the ICD code. When upper respiratory tract infections such as acute pharyngitis, acute tonsillitis, and acute tonsillopharyngitis are considered prediagnoses, five parameters in Table 2 are seen when entering into the system with one of this prediagnosis. In the presence of at least two of these parameters, the system warns the physician that the use of RAT is appropriate. Symptomatic treatment is prescribed when the score is 0–1. If the score is 2–3, RAT is applied. RAT (+) is directed to prescribing antibiotics while for RAT (–), symptomatic treatment is suggested. When the score is 4 and above, the system allows for empirical antibiotic prescription. If RAT is (+) and clinical symptoms are favorable to infection, the system allows use antibiotics according to age group. Swallow and throat swab samples were taken from posterior pharynx and tonsils of patients. The tongue should be pressed with abeslang so that the sample is not mixed with saliva. The test was used as indicated by the company. If clinic symptoms are present but the test is negative, throat culture can be taken in children and adolescents. The advantage of the test is that it takes 5 to 10 min to reach the result. It is a test with a specificity of 95% and sensitivity of 70–90%. 

**Table 1:** Diagnostic codes that can be marked in FMIS for GAS in RAT usage [11].

ICD-10Codes	Diseases
J00	Acute nasopharyngitis [common cold]
J02	Acute pharyngitis
J02.0	Streptococcal pharyngitis
J02.8	Acute pharyngitis due to other specified organisms
J02.9	Acute pharyngitis, unspecified
J03	Acute tonsillitis
J03.0	Streptococcal tonsillitis
J03.8	Acute tonsillitis due to other specified organisms
J03.9	Acute tonsillitis, unspecified

**Table 2:** Modified Centor Criteria based on the usage of RAT [12].

Symptoms	Score
Fever (>38.0)	1
Enlarged and sensitive anterior cervical lymphadenitis	1
Tonsil growth and exudative lesion	1
No coughing	1
Age 3–14 15–44 ≥45	+1 0 –1

### 2.3. Statistical analysis

Descriptive statistics for the obtained data were calculated as numbers and percent frequencies. Differences between years, months, age groups, sexes, antibiotics, and physicians were examined with the Poisson regression model. In statistical evaluations, when P-value was <0.05, the result was accepted significant. SPSS (v. 18) Software was used in the calculations.

## 3. Results

### 3.1. Comparison among years and months

**Figure 1:**
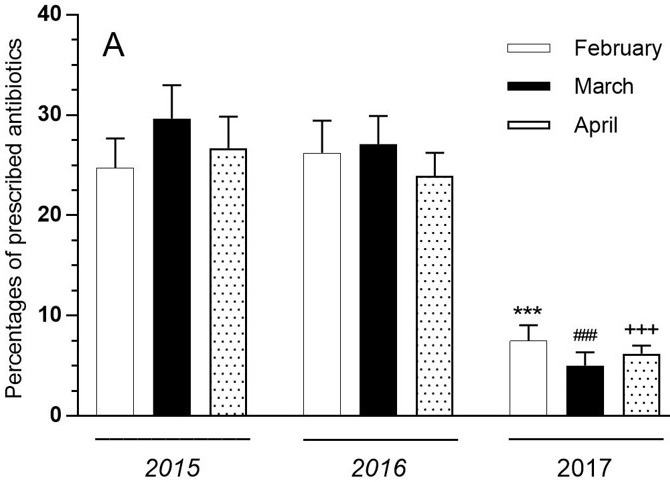

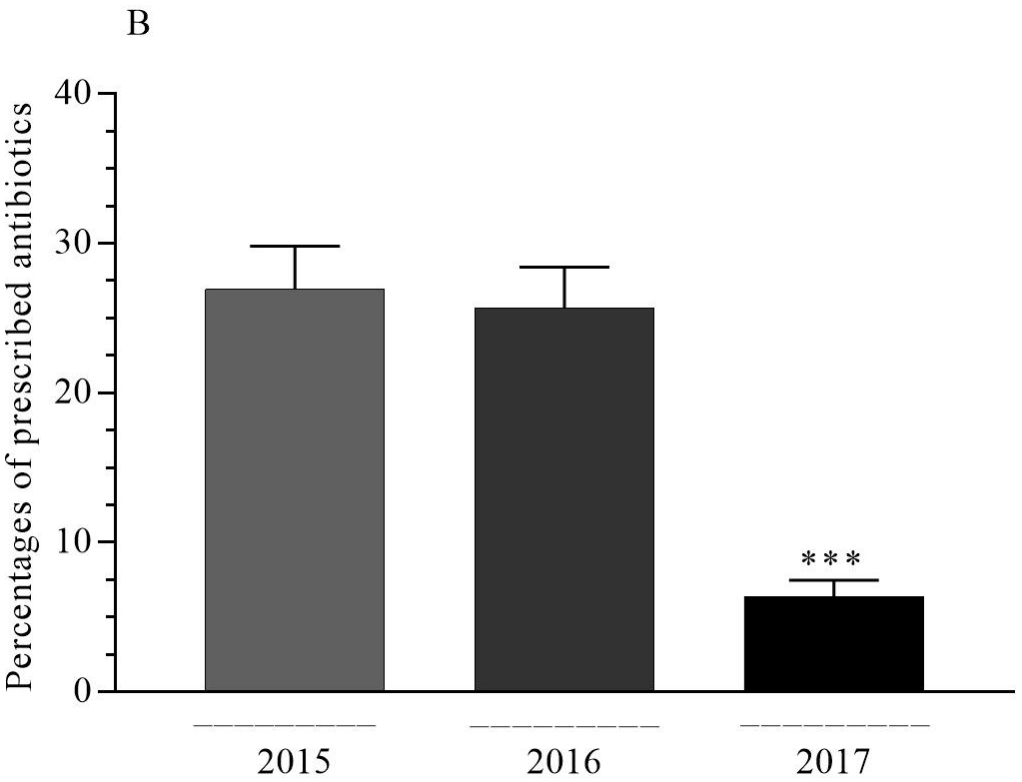
A) Distribution of antibiotic rates according to years and months. ***P < 0.001 compared with February months for 2015 and 2016. ###P < 0.001 compared with March months for 2015 and 2016. +++P < 0.001 compared with April months for 2015 and 2016. Antibiotic prescribing rate is not different between the months over the years. Each value is mean ± SEM. Vertical bars represent SEM. B) Distribution of antibiotic prescriptions of the ten physicians according to years. ***P < 0.001 compared with 2015 and 2016 years. Each value is mean ± SEM. Vertical bars represent SEM.

The differences according to years, months, and sex were investigated for the prescription rates containing antibiotics. There was no significant difference between the months of 2015 and 2016 when family physicians did not use RAT in antibiotic prescriptions. There was a significant difference between the 2015 and 2017 (P < 0.001), and 2016 and 2017 (P < 0.001) in February, March, and April (Figures 1A and 1B). 

There was no significant difference in terms of the antibiotic ratios prescribed in 2015, 2016, and 2017 when men and women were compared. The prescribed antibiotic rates did not show a significant change according to sex. Differences between years were evaluated separately in both sexes; the difference between 2015 and 2017 (P < 0.001), and 2016 and 2017 (P < 0.001) were found statistically significant in both women and men. According to these results, it can be said that antibiotic prescription in both women and men in 2017 has decreased considerably when compared to 2015 and 2016. 

### 3.2. Comparison of age groups for each year

**Figure 2:**
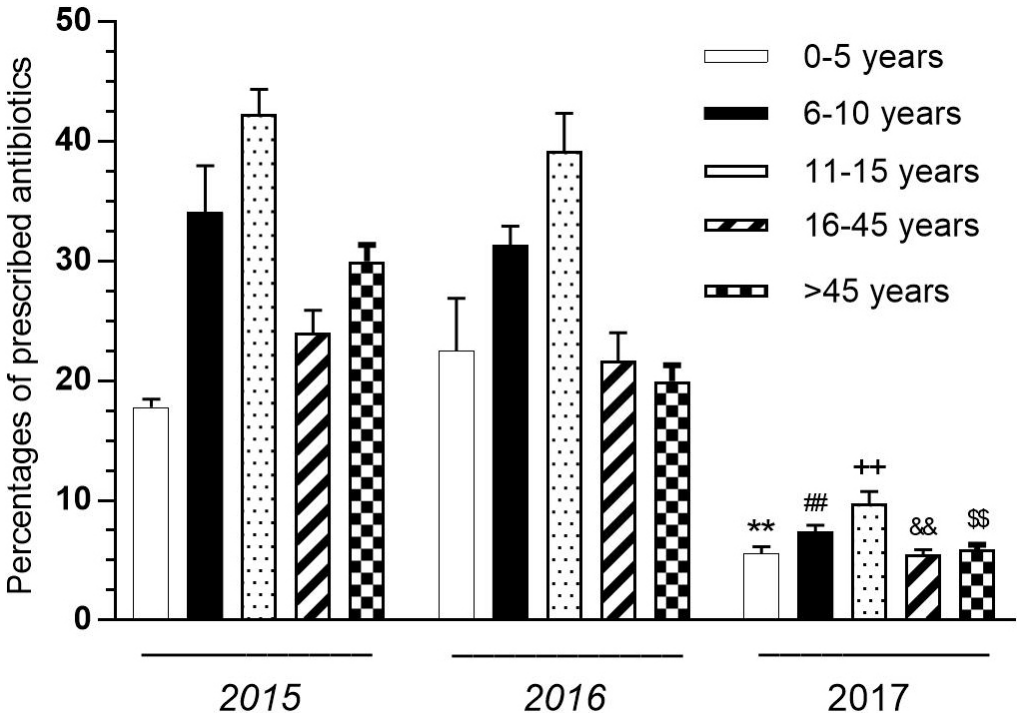
Distribution of prescribed antibiotic rates according to age groups and years. **P < 0.01 compared with 0–5 years for 2015 and 2016. ##P < 0.01 compared with 6–10 years for 2015 and 2016. ++P < 0.01 compared with 11–15 years for 2015 and 2016. &&P < 0.01 compared with 16–45 years for 2015 and 2016. $$P < 0.01 compared with 45 years for 2015 and 2016. Each value is mean ± SEM. Vertical bars represent SEM.

Differences between years were examined in terms of the antibiotic rate prescribed in 0–5, 6–10, 11–15, 16–45, and <45 age groups. According to these results, there was a statistically significant difference in all age groups in 2017 (P < 0.01) (Figure 2). There was no significant difference between 2015 and 2016. 

### 3.3. Comparison of years for each antibiotic

**Figure 3:**
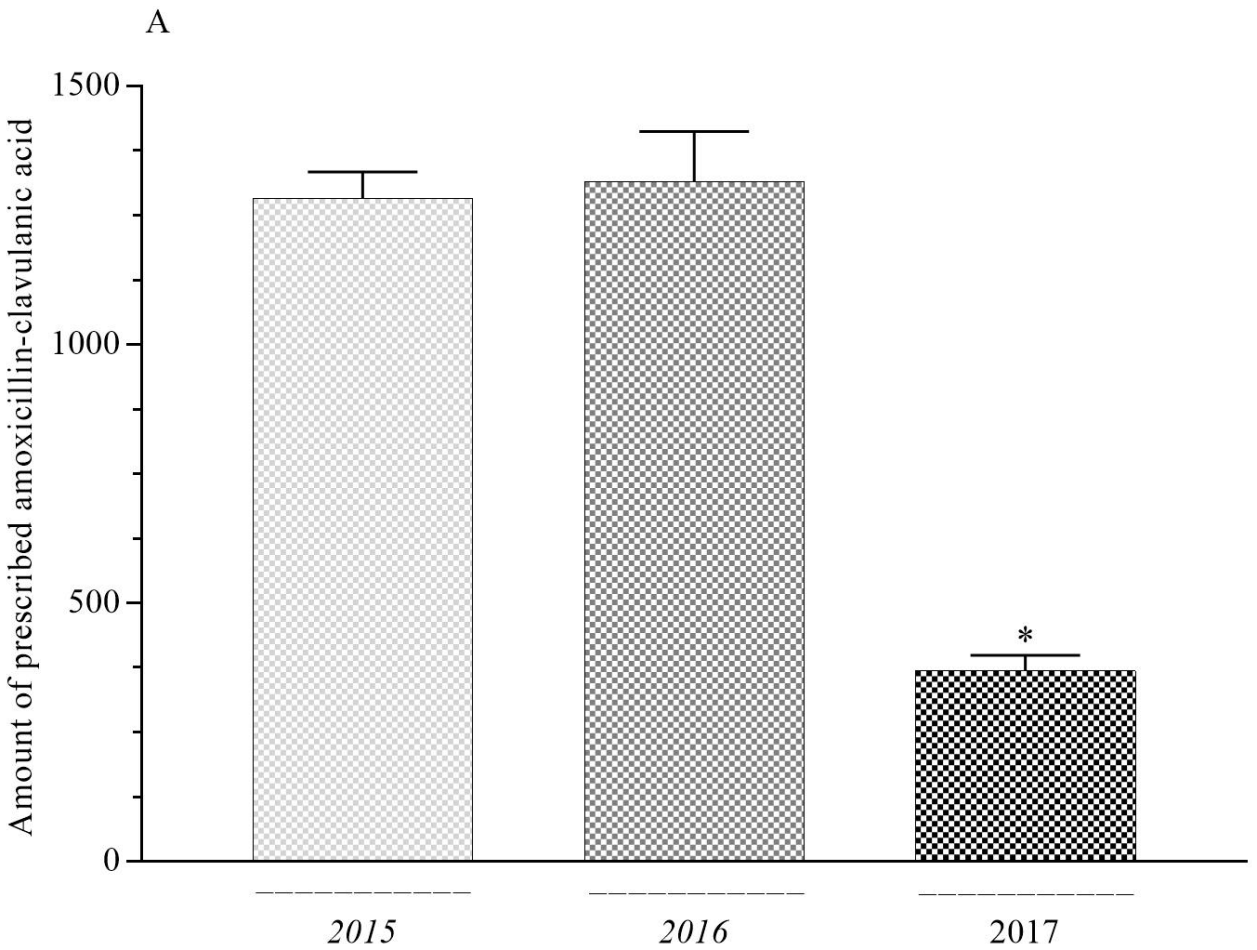

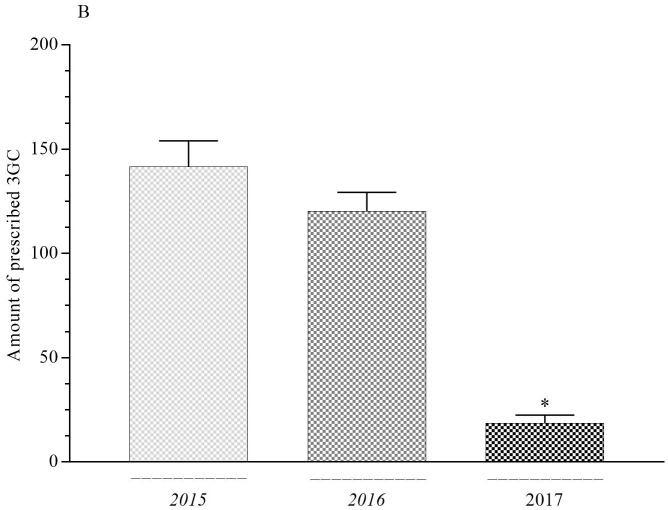

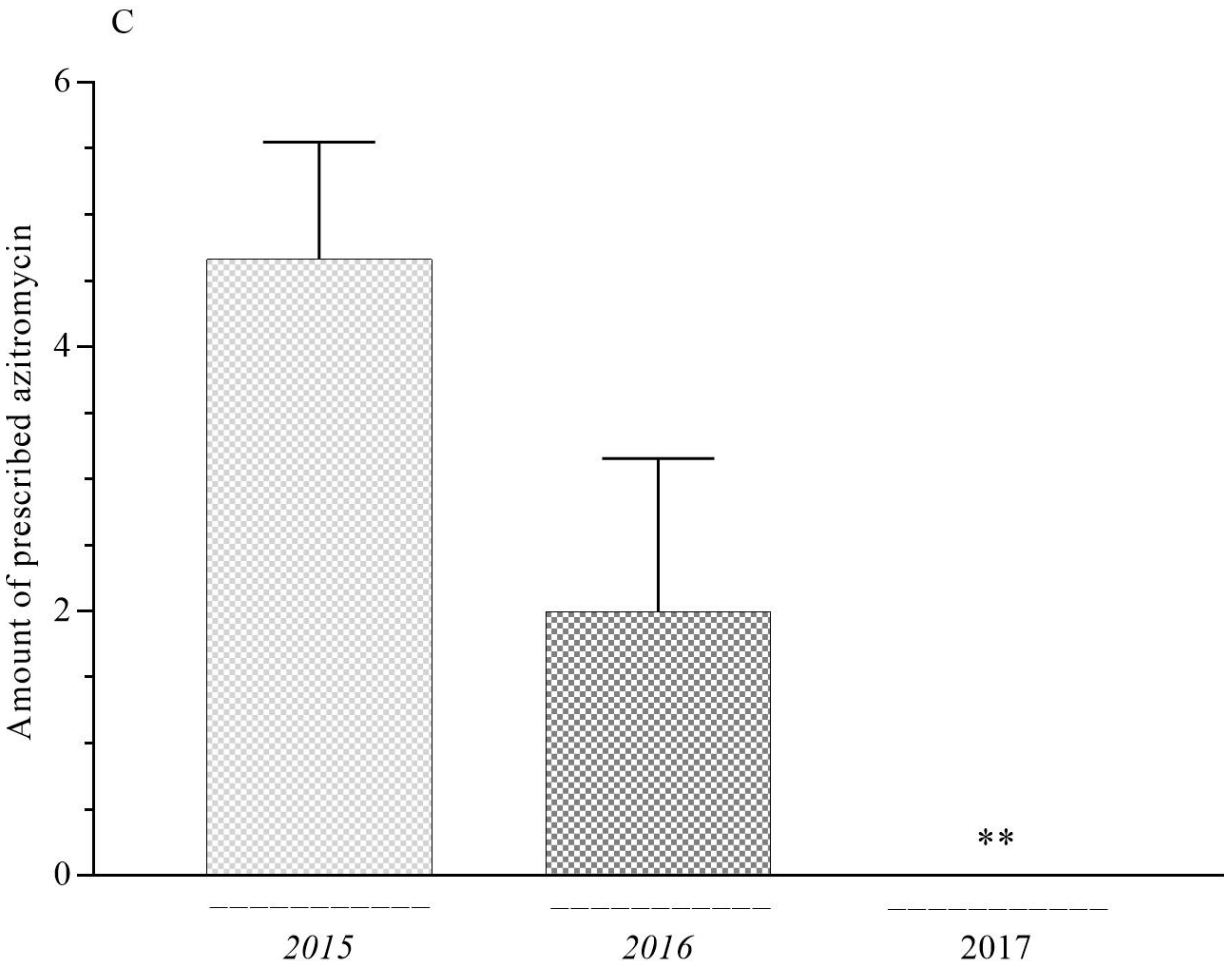

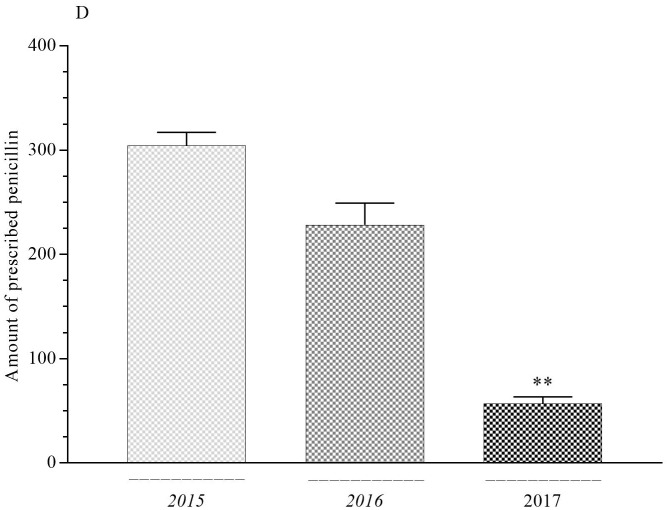
A) Distribution of total antibiotic prescription amount according to years. *P < 0.05 amoxicillin-clavulanic acid compared with 2015 and 2016. Each value is mean ± SEM. Vertical bars represent SEM. B) Distribution of total antibiotic prescription amount according to years. *P < 0.05 3GC compared with 2015 and 2016. Each value is mean ± SEM. Vertical bars represent SEM. C) Distribution of total antibiotic prescription amount according to years. **P < 0.01 Azitromycin compared with 2015 and 2016. Each value is mean ± SEM. Vertical bars represent SEM. D) Distribution of total antibiotic prescription amount according to years. **P < 0.01 Penicillin compared with 2015 and 2016. Each value is mean ± SEM. Vertical bars represent SEM.

The difference between the prescription rate of amoxicillin-clavulanic acid and third-generation cephalosporin (3GC) in 2015 and 2016 and the prescription rate of 2017 was statistically significant (P < 0.05) (Figures 3A and 3B). The difference between the prescription rate of Azithromycin and Penicillin in 2015 and 2016 and the prescription rate of 2017 was statistically significant (P < 0.01) (Figures 3C and 3D). The difference between 2015 and 2016 was not significant for amoxicillin-clavulanic acid, 3GC, azithromycin, or penicillin. The difference between the prescription rate of amoxicillin, second-generation cephalosporin, or clarithromycin was not statistically significant. 

### 3.4. RAT results

**Figure 4:**
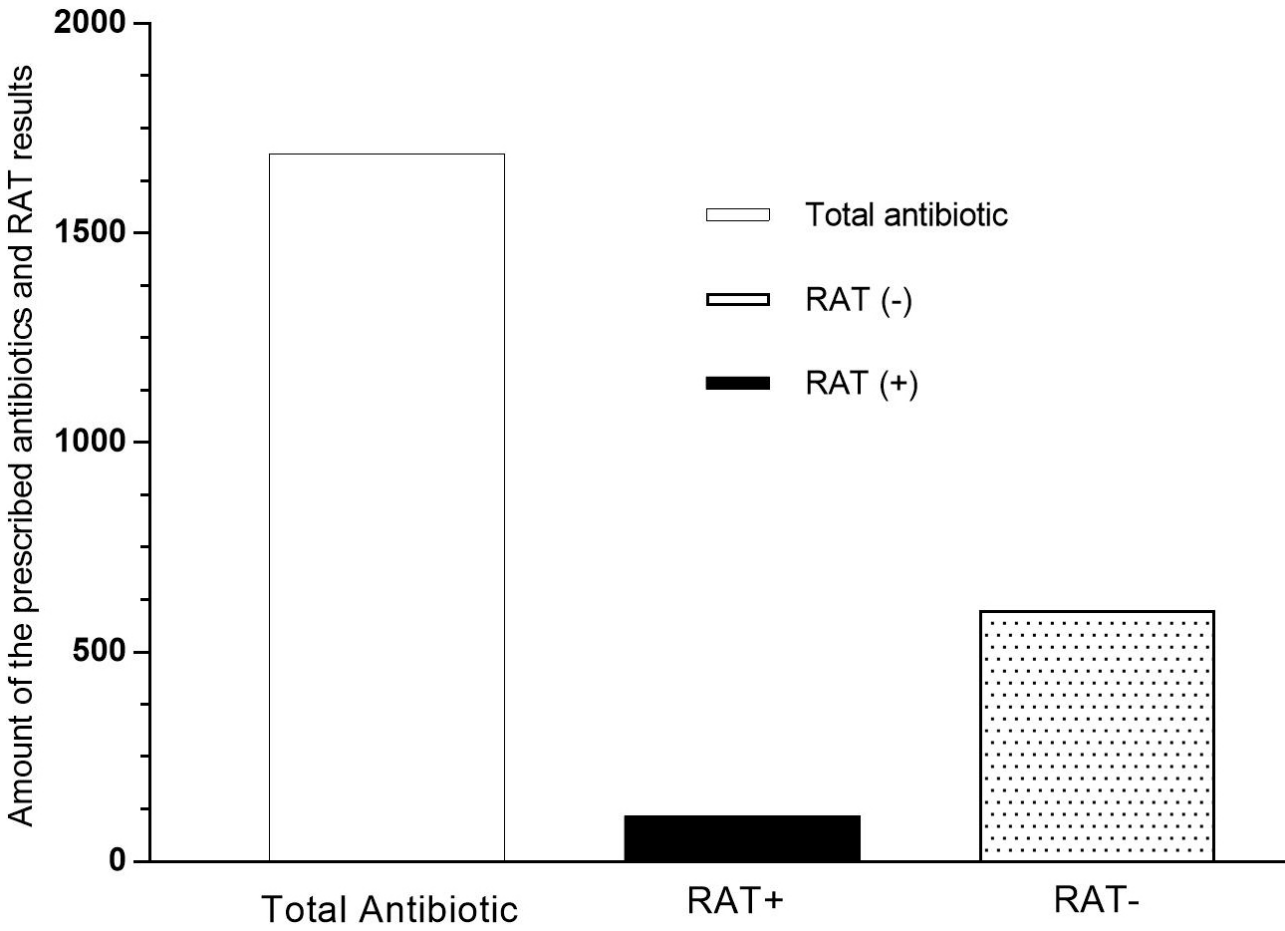
Amount of the antibiotic, RAT (+), and RAT (–) results in 2017. Antibiotic prescribing rate and RAT results are not different between the months over the year.

There was a decrease in the rate of antibiotics prescribed, which is not statistically significant, in 2017 from February to April. The amount of RAT (–) is higher than RAT (+), and antibiotics prescribed are more than the sum of them (Figure 4). Antibiotic prescribing rate and RAT results are not different between the months over the year.

## 4. Discussion

As in many countries, unnecessary or improper drug usage in Turkey is very high due to nonprescribed drug usage and unnecessary prescriptions. Despite all the studies on RDU, there are several important problems such as increased toxic effects of the drugs, drug resistance, treatment failures due to serious adverse effects, and accrued costs. Especially the irrational use of antibiotics is important because it is an obstacle for the treatment of infections and they are used widely. One of the studies on the rational use of antibiotics is to establish a diagnosis for patients with certain diagnostic criteria in upper respiratory tract infections (URI) by using RAT and prescribing antibiotics if they are necessary. The Ministry of Health provided training for RAT use to primary care physicians in January 2017. Then in February 2017, RAT was performed in the patient groups which were selected according to the modified Centor criteria. In this study, RAT applications were evaluated in 10 family medicine units, which are considered to be a sample of many sections of the society such as city center, town, hospital, or remote area and slum area in order to provide homogenization for the population of 35,000 patients in Kırıkkale Province. The changes in the total antibiotic prescribing numbers of the family physicians, who participated in the study in the months of February, March, April 2015 and 2016 were examined. 

The effect of using RAT in Spain between January and May was examined in 543 patients aged 14 or older with clinical findings of acute pharyngitis and who were evaluated according to the Centor scoring. There was a significant difference in prescribing antibiotics between the control (281 patients) and study (262 patients) groups; it was 60% in the control group without RAT and reduced to 26.9% in the study group that applied RAT [13]. Madurell et al. [14] showed that for the 276 adult patients with acute pharyngitis who participated in the study, the antibiotic prescription ratio was 85% in the control group, while it was 75% in the study group. The use of RAT was also investigated in terms of complications, antibiotic replacement, and patient satisfaction. It was reported that the side effects, complications, and antibiotic resistance which may develop due to antibiotic use can be prevented by increasing the patient satisfaction, then a decrease in antibiotic prescription can be ensured [15–17]. The selection of family physicians from different residential areas has enabled the provision of homogenization for parameters like the number of patients admitted to the clinic, easy or difficult access to the treatment, expectation levels from the physician [18,19]. Thus, different socio-cultural and socio-economic patient populations in the same province were included in this study. As the main purpose of the study was to reach the number of antibiotics prescribed, patients were taken into consideration in the same period of disease, with similar diagnosis, short term (1–3 days) of other antibiotics, and the number of antibiotics in this prescription. Efficacy and treatment time were not considered.

When the suitability of the indications for drugs prescribed worldwide is examined, it is stated that the rate of antibiotics prescribed based on the RDU principles is significantly low. Diagnosis with acute tonsillopharyngitis is also present among the prescriptions that contain the most problems [20]. In our study, the aim of selecting a topic for patients with URIs is that both the excess of the unnecessary antibiotic prescribing, as well as the fact that a test entity (RAT) that verifies the diagnosis may have an effect on the outcome.

It has been mentioned that pharyngitis or tonsillopharyngitis caused by GAS in humans may cause some undesirable conditions when not treated. Although throat culture is considered to be the de gold standard for GAS detection, the result can be reached at the earliest after 24 h. Especially in family medicine policlinics where policlinic services are in a large number, RAT, which is a practical and reliable test, accelerates the diagnosis, provides a great ease by giving results in 5 min [21]. In order to compare the results of throat culture with RAT, throat culture was also requested. Throat culture sampling and evaluation process, which was planned to control the reliability of RAT, was not fully practicable in intensive outpatient settings. While RAT is negative, the result is completely negative in a certain number of patients whose throat culture was requested. This has been evaluated as a result that supports the reliability of RAT. It has been reported that 505 pediatric patients presenting with the complaint of URI; 86% were RAT-negative, 14% were RAT-positive, 84% throat culture-negative and 16% were positive for throat culture. The number of patients who were evaluated as both culture and RAT negative was 84% and the number of patients evaluated as culture and RAT positive was 13%. The number of patients with culture and RAT inconsistency was 3% [22]. 

Despite the decrease in the number of prescribed antibiotics in 2017 compared to 2015 and 2016, the number of antibiotics prescribed leads to a difference in the number of RAT positive and RAT negative patients, because it is predicted that the antibiotic can be empirically written to the system according to the Modified Centor Score. However, the use of the mentioned scoring method provided a substantial decrease in antibiotic prescriptions compared to 2015 and 2016. It was observed that the trainings for the drug prescribing procedures for physicians as part of RDU in recent years can make a positive impact on the results [23]. Studies on public awareness show that the number of patients demanding antibiotics from physicians reduced. The contribution of education to the whole society, especially to physicians, is evident in this study. Not only the rational use of antibiotics, but also the rational use approach in all other drug groups can be increased by the training of physicians and society [24–26].

Feedback from physicians suggests that RAT positively affected the diagnosis of GAS, antibiotic prescribing, and more self-confident behavior in the face of the patient in the case of URIs. The use of RAT in fast and reliable detection of other microorganisms, not only GAS, will support this condition. It has been determined that the results of the use of RAT for Haemophilus Influenzae in Korea in 2011–2012 are similar to those of RAT for GAS [27]. The physicians who participated in our study also agreed that the continuity of RAT applications during the outpatient clinic would be positive for the future. RAT-negative results were higher in 2017 than RAT-positive. This result shows that the decrease in antibiotic prescription in 2017 may be related to the use of RAT. Increased use of RAT indicates that antibiotic prescribing may be further reduced.

While developing strategies for health systems, it is important to use the capital allocated to health in a rational way by taking into consideration the increase in treatment options, population growth, and technological developments. When allocating budget for health expenditures, the economic impacts of treatment should be taken into consideration as well as efficiency and safety assessments. Unconsciousness in the use of antibiotics in Turkey is an important problem. This is a burden on health expenditures and is a major problem in the underdeveloped countries, but it also poses a problem in developed countries [26,28,29]. Our study reveals the importance of both the use of RAT and the training of physicians and community in the scope of RDU in terms of antibiotic prescribing.

To counclude, in our study, there was no significant difference in the number of antibiotic prescriptions in the URIs group between 2015 and 2016, whereas the year 2017 when rapid antigen test (RAT) was used was significantly different. Extending the use of RAT not only for group a streptococcus (GAS) but also for other microorganisms will be important for use as a practical, rapid, and reliable diagnostic method in centers providing intense polyclinic services. More simple methods providing treatment options in a shorter time, which may have better results in terms of sensitivity and specificity in GAS tonsillopharyngitis, should continue to be investigated. The development of advanced diagnostic methods for the differentiation of acute GAS infection from chronic carriage will contribute to the rational use of antibiotics and will provide benefits to the national economy in terms of pharmacoeconomic aspects.
